# Is increased colorectal screening effective in preventing distant disease?

**DOI:** 10.1371/journal.pone.0200462

**Published:** 2018-07-12

**Authors:** Gaius Julian Augustus, Denise J. Roe, Elizabeth T. Jacobs, Peter Lance, Nathan A. Ellis

**Affiliations:** 1 Cancer Biology Graduate Interdisciplinary Program, Tucson, AZ; 2 University of Arizona Cancer Center, University of Arizona, Tucson, AZ, Tucson, AZ; University Hospital Llandough, UNITED KINGDOM

## Abstract

**Background:**

Screening in the average risk population for colorectal cancer (CRC) is expected to reduce the incidence of distant (i.e., metastatic) CRCs at least as much as less advanced CRCs. Indeed, since 2000, during which time colonoscopy became widely used as a screening tool, the overall incidence of CRC has been reduced by 29%.

**Objective:**

The purpose of the current study was to determine whether the reduction of incidence rates is the same for all stages of disease.

**Methods:**

We evaluated incidence data from the Surveillance, Epidemiology, and End Results (SEER) program from 2000–2014 for Localized, Regional, and Distant disease. Joinpoint models were compared to assess parallelism of trends. Data were stratified by race, age, tumor location, and sex to determine whether these subgroupings could explain overall trends.

**Results:**

Inconsistent with the expectations of a successful screening program, the reduction in incidence rates of distant CRCs from 2000–2014 has been slower than the reductions in incidence rates of both regional and localized CRCs. This trend is evident even when the data are stratified by age at diagnosis, sex, race, or tumor location.

**Conclusions:**

The slower decrease in the incidence rate of distant disease is not consistent with a screening effect, that is, CRC screening may not be effective in preventing many distant CRCs. As a consequence, distant CRCs represent an increasing fraction of all CRCs, accounting for 21% of all CRCs in 2014. The analysis indicates that inadequate screening does not explain the slower decrease in incidence of distant CRCs. Consequently, we suggest that a subtype of CRC exists that advances rapidly, evading detection because screening intervals are too long to prevent it. Microsatellite unstable tumors represent a known subtype that advances more rapidly, and we suggest that another rapidly advancing subtype very likely exists that is microsatellite stable.

## Introduction

Colorectal cancer (CRC) remains the second most common fatal malignancy after lung cancer in the United States and other developed countries [1]. The prevailing concept for the formation of sporadic CRCs is that most of them develop over a period of 7 to 15 years from benign adenomatous polyps (adenomas) [2]. In the United States, screening for CRC by fecal blood test, flexible sigmoidoscopy, or colonoscopy is recommended for all individuals between the ages of 50 and 75 years [3]. Screening by colonoscopy is recommended every 10 years, with 3–5 year follow-up if an adenoma is detected. Positive results from a fecal blood test or flexible sigmoidoscopy are verified by colonoscopy. The rationale for CRC screening is that (i) asymptomatic adenomas can be detected and removed before ever progressing to cancer, and (ii) histological stage at the time of treatment is the most predictive marker of long-term prognosis. Assuming CRC is indeed a slow-growing malignancy, current screening guidelines should be effective in meeting these two criteria.

A successful CRC screening program is one that decreases incidence rates of CRC by adenoma removal and that shifts the burden of invasive disease at the time of diagnosis from more advanced (i.e., regional and distant/metastatic) tumors to less advanced (localized) tumors, which are more readily cured by surgical resection. Since 2000, the quality of colonoscopy has improved and an increasing percentage of US persons have adopted CRC screening [4,5]. In support of screening efficacy, fecal blood testing and colonoscopy, respectively, have been reported to reduce CRC mortality by as much as 32% [6] and 68% [7]. Consistent with the increased adoption of CRC screening since 2000, the overall incidence of CRC has decreased 29% from 2000–2014 (SEER program, Nov 2016 submission).

**Fig 1:**
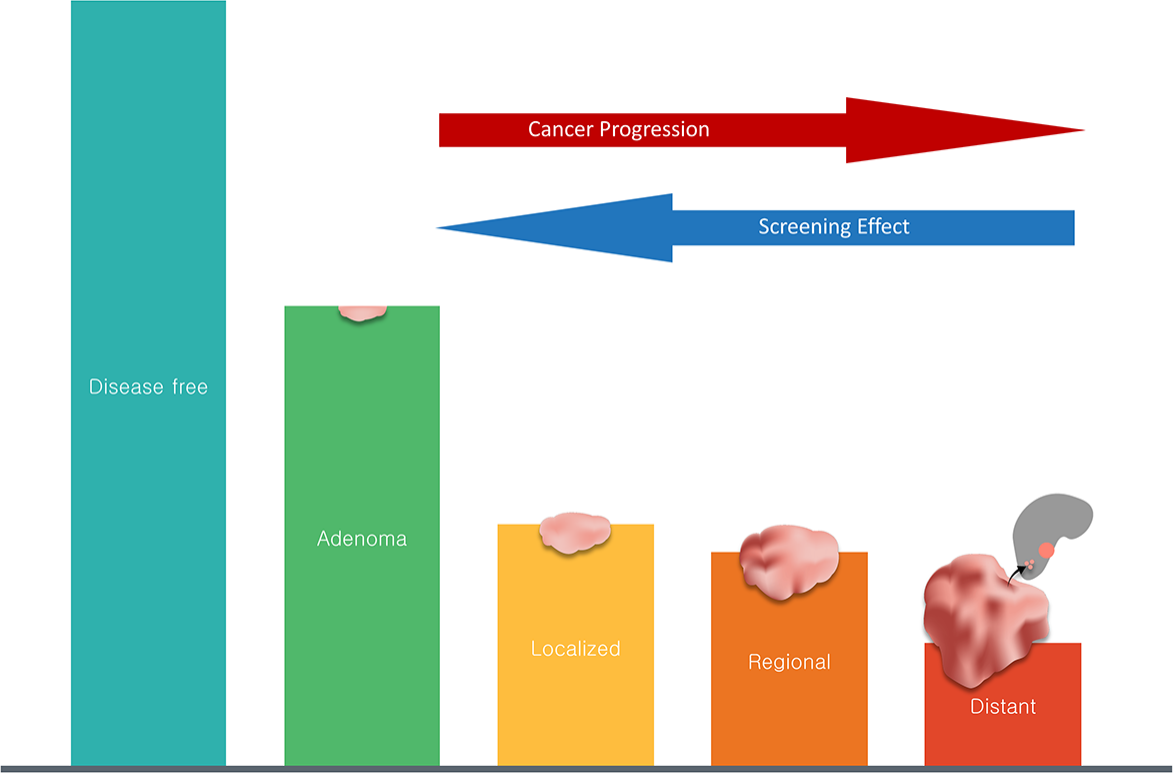
Model of a successful screening program. The widely accepted concept is that colonic neoplasia progress from less advanced to more advanced stages (from adenoma to localized to regional to distant) and become more symptomatic as they progress. As screening quality is improved and more people are being screened, as has been the case in the US since 2000, we expect a screening effect that reduces the incidence rates of all stages of disease. This model predicts that a successful screening program would exert its largest reduction in incidence on distant CRC.

The primary objective of our study was to determine whether the reduction in CRC incidence has been equal for all stages of disease. The prevailing concept is that CRCs progress from adenoma to localized cancer to regional to distant disease. Consequently, detection and removal of early lesions through screening should decrease the incidence of later-stage disease (Fig 1). By this reasoning, distant CRCs should show the largest reduction in incidence. Here, we analyzed incidence rate data from the Surveillance, Epidemiology, and End Results (SEER) program and present evidence of a disturbing trend: from 2000–2014 the incidences of localized and regional CRC have decreased substantially whereas the incidence rate of distant (i.e., metastatic) CRC has not.

## Materials and methods

### Data acquisition

We used data from the National Cancer Institute (NCI) Surveillance, Epidemiology, and End Results (SEER) Program database. Incidence rate data queries were made to the SEER 18 (SEER 18 Regs Research Data + Hurricane Katrina Impacted Louisiana Cases, Nov 2016 Sub (2000–2014) <Katrina/Rita Population Adjustment>—Linked To County Attributes—Total U.S., 1969–2015 Counties) registry data using SEER*Stat v8.3.4. This database includes data from 18 regions from 2000 to 2014. From the full SEER dataset, we restricted our case definition to patients with cancers of the colon or rectum (Site recode ICD-O-3/WHO 2008). All age-adjusted incidence rate data were expressed as cases per 100,000 as calculated from the 2000 US standard population. Stage data–Summary stage 2000 (1998+)–defines stage as **L**ocalized, **R**egional, **D**istant, or Unknown/unstaged. Definitions for this model of staging can be found at the SEER website (https://seer.cancer.gov).

### Statistical analyses

To evaluate the data for significant linear trends, we used the Joinpoint Regression Program v4.5.0.1 (Joinpoint Regression Program, Version 4.5.0.1—June 2017; Statistical Methodology and Applications Branch, Surveillance Research Program, National Cancer Institute). We stratified by age of diagnosis (Early: < 50; Middle: 50–64; Late: 65+), by race (American Indian/Alaska Native, Asian or Pacific Islander, Black or African American, White), by tumor location (Proximal: cecum, appendix, ascending colon, hepatic flexure, and transverse colon; Distal: splenic flexure, descending colon, sigmoid colon, rectum, and rectosigmoid junction), and by sex (Male, Female). We also used comparability tests to compare trends in regional vs. distant disease and localized vs. distant disease. The test of parallelism determined whether trends of regional and distant CRC and localized and distant CRC were parallel over given time periods, again using p < 0.05 as an indicator of non-parallel trends.

Statistics were conducted in Joinpoint Regression Program, and figures were produced in R version 3.3.2 (dplyr v0.5.0, ggplot2 v2.2.1, scales v0.4.1, readr v1.0.0, cowplot v0.8.0, and all dependencies).

### Availability of data and material

All data that support the findings of this study are publicly available from SEER (http://seer.cancer.gov) using SEER*Stat. Minimal data for reproducibility and all code used in the processing of the data and visualizations, input for and output of the Joinpoint Regression Program, and tables generated for analysis during the current study are available in the gaiusjaugustus/DistantCRCRates repository on GitHub. Code at the time of submission is available at the following release: https://github.com/gaiusjaugustus/DistantCRCRates/releases/tag/20180413 (doi:10.5281/zenodo.1218098).

## Results

### Decrease in incidence rate of distant CRC is slower than the decrease in incidence rates of localized and regional CRC

**Fig 2:**
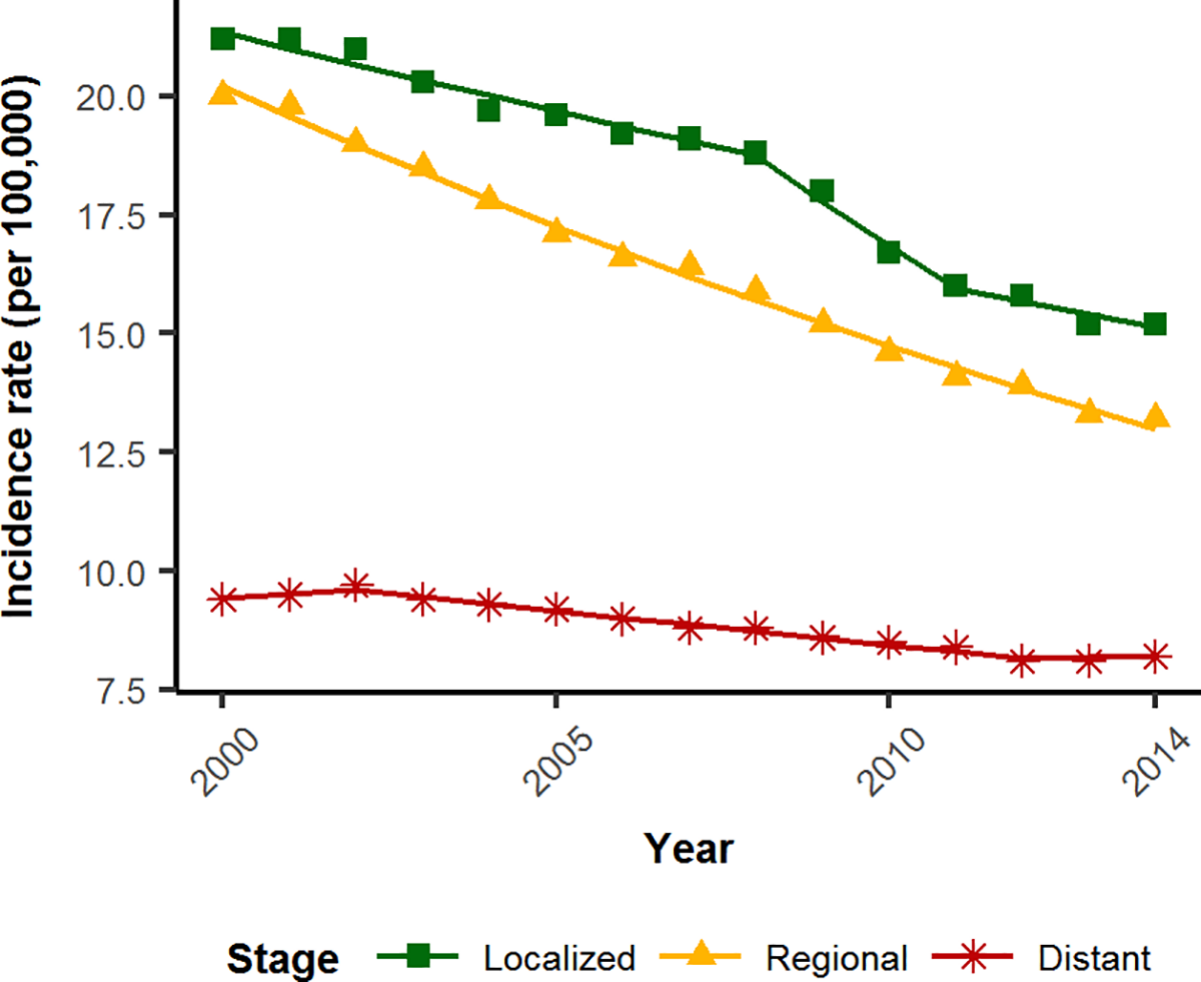
The incidence rate of distant CRC is decreasing much more slowly than non-distant disease. Green squares, localized; yellow triangles, regional; red stars, distant. Incidence rates are expressed per 100,000.

Since 2000, CRC incidence for the overall US population has seen a reduction of 2.3% per year from 2000–2008 and 4.6% per year from 2008–2011, and it has been stable from 2011–2014 [annual percentage change (APC) = -1.3]. In 2000, incidence rates of localized, regional, and distant CRC were 21.22, 19.98, and 9.44 cases per 100,000, respectively (Fig 2). In 2014, the rates of localized and regional CRC had decreased, respectively, by 6 points to 15.23 (a 28.3% reduction) and by 6.8 points to13.19 (a 34% reduction). However, distant CRC incidence rates had only decreased by 1.2 points to 8.24 (a 12.8% reduction). In 2000, localized, regional, and distant CRC accounted for 39%, 36.8%, and 17.3% of CRCs, respectively. In 2014, localized had remained a stable proportion of CRCs (39.2%), regional had decreased to 34%, and distant CRCs had increased to 21.1% of all CRCs. The remaining proportion of disease is unstaged CRC and was omitted from further analysis.

In order to determine whether or not the decrease in regional disease differed from that of distant disease, we performed a comparison analysis of joinpoint models (Table 1). Regional CRC decreased by 3.10% per year (APC = -3.10, p < 0.001). Distant CRC was statistically stable from 2000–2002 (APC = 0.92, p = 0.24) before incidence began to decline in 2002. Incidence declined by 1.6% per year from 2002 to 2012 (APC = -1.60, p < 0.001), then remained statistically stable from 2012 to 2014 (APC = 0.2, p = 0.8). A pairwise comparison of the two joinpoint regressions indicated that the trends of regional and distant CRC are statistically different from each other (p < 0.001), with distant CRC decreasing at a significantly slower rate than regional CRC.

**Table 1:** Summary of localized, regional, and distant joinpoint models.

	Localized			Regional			Distant			Pairwise Comparison	
	Year	APC	p-value	Year	APC	p-value	Year	APC	p-value	Localized/Distant	Regional /Distant
Overall population	2000–2008	-1.6	< 0.001	2000–2014	-3.10	< 0.001	2000–2002	0.92	0.24	< 0.001	< 0.001
	2008–2011	-5.22	0.01				2002–2012	-1.61	< 0.001		
	2011–2014	-1.80	0.07				2012–2014	0.18	0.81		
Age of diagnosis											
Middle diagnosis (50–64)	2000–2014	-1.25	< 0.001	2000–2011	-2.36	< 0.001	2000–2014	-0.44	0.004	0.007	< 0.001
				2011–2014	0.74	0.52					
Late diagnosis (65+)	2000–2007	-2.28	< 0.001	2000–2008	-3.86	< 0.001	2000–2002	0.24	0.90	< 0.001	< 0.001
	2007–2014	-5.01	< 0.001	2008–2014	-4.96	< 0.001	2002–2014	-2.83	< 0.001		
Tumor Location											
Proximal	2000–2008	-0.26	0.50	2000–2014	-3.39	< 0.001	2000–2014	-1.56	< 0.001	0.029	< 0.001
	2008–2011	-4.97	0.16								
	2011–2014	-0.33	0.85								
Distal	2000–2007	-2.44	< 0.001	2000–2014	-2.88	< 0.001	2000–2002	1.83	0.13	< 0.001	< 0.001
	2007–2014	-4.16	< 0.001				2002–2012	-1.71	< 0.001		
							2012–2014	1.20	0.27		
Race											
Black/African American	2000–2007	0.36	0.40	2000–2014	-3.39	< 0.001	2000–2012	-1.32	< 0.001	0.022	< 0.001
	2007–2014	-3.85	< 0.001				2012–2014	-6.02	0.05		
White	2000–2008	-1.91	< 0.001	2000–2014	-3.01	< 0.001	2000–2002	1.09	0.18	< 0.001	< 0.001
	2008–2011	-5.67	0.015				2002–2012	-1.62	< 0.001		
	2011–2014	-1.37	0.18				2012–2014	0.99	0.21		
Native American or Alaska Native	2000–2014	-1.4	0.057	2000–2014	-1.62	0.02	2000–2014	0.39	0.58	0.083	0.10
Asian or Pacific Islander	2000–2008	-0.6	0.23	2000–2014	-3.67	< 0.001	2000–2014	-1.52	< 0.001	0.296	0.001
	2008–2014	-3.76	< 0.001								
Sex											
Female	2000–2008	-1.4	< 0.001	2000–2014	-3.07	< 0.001	2000–2014	-1.36	< 0.001	< 0.001	< 0.001
	2008–2011	-4.22	0.038								
	2011–2014	-1.63	0.096								
Male	2000–2008	-1.95	< 0.001	2000–2014	-3.23	< 0.001	2000–2014	-1.33	< 0.001	< 0.001	< 0.001
	2008–2011	-6.18	0.02								
	2011–2014	-2.04	0.096								

Summary of localized, regional, and distant joinpoint models for the general population as well as stratified by age of diagnosis, tumor location, race, and sex. Each segment is given as a year range with the estimate of the annual percent change (APC) and the p-value associated with the APC. Pairwise comparison p-values (testing for parallel trends) of regional and distant are given for each stratum.

To determine whether the incidence rate of distant disease was also less than localized disease, we again compared joinpoint models. Localized CRC decreased 1.6% per year from 2000–2008 (APC = -1.6, p < 0.001), decreased 5.22% from 2008–2011 (APC = -5.22, p = 0.01) and decreased 1.8% from 2011–2014 (APC = -1.8, p = 0.07). The two trends of localized and distant disease were also not statistically parallel (p < 0.001), again with distant CRC decreasing at a significantly slower rate than localized CRC.

### Decrease in incidence rate of distant CRC is slower in patients diagnosed with CRC at 50 or more years of age

**Fig 3:**
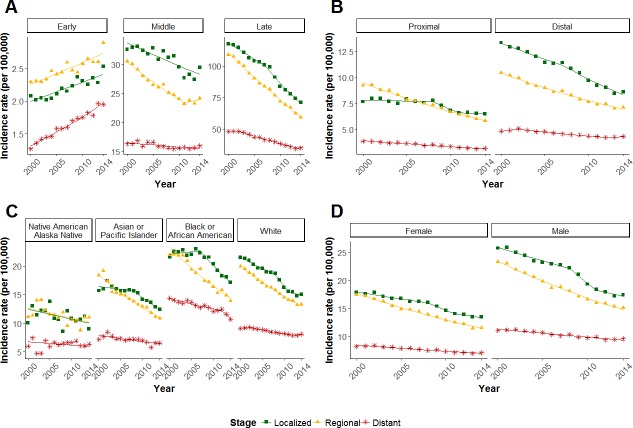
Incidence rates of distant, regional, and localized CRC by age, sex, site, and race. Change in the incidence rates (per 100,000) of CRC by stage stratified by (A) age (<50, 50–64, 65+), (B) site (distal, proximal), (C) race (Native American/Alaskan Native, Asian or Pacific Islander, Black or African American, and White), and (D) sex. Green squares, localized; yellow triangles, regional; red stars, distant.

Because persons under 50 years old only screen for CRC if they have a first-degree relative with CRC, which comprises less than 20% of the population, we compared incidence rates of persons younger than 50 with persons 50–64 years old and persons 65 years and older. As has been reported previously [8], we found that early-onset CRCs, defined as CRCs diagnosed before age 50, are more likely to present at more advanced stages compared to CRCs from patients 50 or more years of age. Fig 3A shows that the rate of distant early-onset CRCs is increasing more rapidly than localized or regional early-onset CRCs (Table 2, localized vs distant, p < 0.001; regional vs distant, p < 0.001). From 2000–2014, localized and regional early-onset CRC incidence rates increased by 1.39% and 1.33% per year (localized, APC = 1.39, p < 0.001; regional, APC = 1.33, p < 0.001), respectively. During the same time, the incidence rate of distant CRC increased by 2.9% per year (APC = 2.90, p < 0.001), over two times the percentage increase as localized and regional early-onset CRC.

**Table 2:** Summary of localized, regional, and distant joinpoint models for early-onset CRC.

	Localized			Regional			Distant			Pairwise Comparison	
	Year	APC	p-value	Year	APC	p-value	Year	APC	p-value	Localized/Distant	Regional /Distant
Early diagnosis (< 50)	2000–2014	1.39	< 0.001	2000–2014	1.33	< 0.001	2000–2014	2.90	< 0.001	< 0.001	< 0.001

Summary of localized, regional, and distant joinpoint models for the early onset patients (< 50 years of age at diagnosis). Each segment is given as a year range with the estimate of the annual percent change (APC) and the p-value associated with the APC. Pairwise comparison p-values (testing for parallel trends) of regional and distant are given.

In contrast, in both age groups diagnosed with CRC at 50 or more years of age (middle: 50–64 years; late: 65+ years), incidence rates for all three stages decreased from 2000–2014. As seen with the unstratified data, the decrease in incidence rate of distant disease was significantly slower compared both to the decrease in incidence rate of regional disease (middle, p < 0.001; late, p < 0.001) and to the decrease in incidence rate of localized disease (middle, p = 0.007; late, p < 0.001).

### Decrease in incidence rate of distant CRC is slower for both proximal and distal CRCs

To address whether tumor location might explain the slower reduction of distant CRCs, we stratified joinpoint models by distal and proximal CRC (Fig 3B, Table 1). In proximal CRCs, from 2000–2014, the incidence rate of distant disease decreased annually by 1.56% (p < 0.001), whereas the incidence rate of regional disease decreased annually by 3.39% (p < 0.001). Localized disease was relatively stable throughout this period (Table 1). In distal CRCs, the incidence rate of distant disease was stable from 2000–2002 (APC = 1.83, p = 0.13), then it decreased annually by 1.71% from 2002–2012 (p < 0.001), and it was again stable from 2012–2014 (APC = 1.2, p = 0.27). The incidence rate of regional disease in distal tumors decreased by 2.88% from 2000–2014 (APC = -2.88, p < 0.001) and the incidence rate of localized disease decreased annually by 2.44% from 2000–2007 (p < 0.001) and by 4.16% from 2007–2014 (p < 0.001).

Thus, over the time period from 2000–2014, incidence rate models for distant CRC and regional CRC were statistically different for both distal and proximal disease (p < 0.001 for both comparisons). Similarly, incidence rate models for distant CRC and localized CRC were statistically different for distal disease (p < 0.001) and for proximal disease (p = 0.029). In all cases, distant CRC incidence rates decreased significantly more slowly than non-distant disease.

### Decrease in incidence rate of distant CRC is slower for most ethnic groups

Because non-white ethnic groups have lower rates of colorectal cancer screening than whites [9,10], to address whether differences in the frequency of screening might explain the slower reduction of distant CRCs, we stratified joinpoint models by race.

African Americans have the highest CRC incidence rates of all ethnic groups in the US and are also more likely to be diagnosed at later stages [11]. This observation is reflected in Fig 3C, where incidence rates of distant CRC are higher than rates in other races over the same period of time. The lower rates of CRC screening in the African American population might be expected to result in more comparable decreases in rates in distant and earlier-stage disease; however, joinpoint models for distant CRC in African Americans compared both to regional and to localized CRC in African Americans were statistically different, that is, the incidence rate of distant CRC decreased more slowly than the incidence rates of less advanced tumors (Table 1; localized vs. distant, p = 0.022; regional vs. distant, p < 0.001). For Asians, the incidence rate of distant CRC decreased more slowly than the incidence rate of regional (p = 0.001), whereas the decrease in the incidence rate for localized disease was not statistically different from that for distant disease. For Native Americans, joinpoint models of all stages were statistically similar (localized vs. distant, p = 0.083; regional vs. distant, p = 0.10). The smaller population sizes underpinning the data from the Native American population make rate comparisons less certain.

### Decrease in incidence rate of distant CRC is slower in both males and females

To address whether sex might explain the slower decrease in incidence rate of distant disease compared to that of earlier-stage disease, we stratified the data by sex (Fig 3D). The incidence rate of distant disease decreased by 1.36% and 1.33% each year for females (APC = -1.36, p < 0.001) and males (APC = -1.33, p < 0.001), respectively, whereas the incidence rate of regional disease decreased annually by 3.07% for females (p < 0.001) and annually by 3.23% for males (p < 0.001). In females, localized CRC decreased annually by 1.4% from 2000–2008 (p = 0.001) and by 4.22% from 2008–2011 (p = 0.038), then it was stable from 2011–2014 (APC = -1.63, p = 0.096). For males, localized disease decreased annually by 1.95% (p = 0.001) from 2000–2008 and by 6.18% from 2008–2011 (p = 0.02), then it was stable from 2011–2014 (APC = -2.04, p = 0.096). Overall, the joinpoint models of distant CRC differed from those of regional and localized CRC for both males and females (Table 1; p < 0.001 for all comparisons).

## Discussion

Analysis of incidence data for CRC from the SEER program over the years 2000–2014 shows that the overall incidence rate of CRC has decreased by 29.7%, an outcome ascribed by some to an increase in CRC screening in the general population. Paradoxically, the reduction in incidence has not been evenly distributed over all stages of disease at diagnosis. Overall, the incidence rate of distant disease, which is predominated by CRC that has metastasized to the liver, has decreased by 12.8%, whereas the incidence rate of regional and localized disease has decreased by 34% and 28%, respectively. Our analysis has shown that these differences in incidence rates are statistically significant, with the decrease in distant disease being slower than the decrease in regional and localized disease.

In consideration of the impact of these epidemiologic trends, it is first important to acknowledge that these differences in percentage change are not small. It is unlikely that they will be explained by small differences in epidemiologic variables. Secondly, although joinpoint modeling suggests that rates of change can vary over different periods in the 2000–2014 time, the conclusions from joinpoint models are not substantially different from the conclusions drawn from linear models (analysis not shown). Thirdly, in persons less than 50 years old—a population subgroup that is predominantly not screening—the incidence rates of CRC are increasing for all stages [12], but the incidence rate of distant CRC is increasing faster than localized and regional. Finally, we found that profound differences in percentage change between distant and non-distant CRC were present in every subgroup in which it was possible to stratify the data, namely, age, sex, race, and tumor location. Consequently, these differences cannot be ascribed to variables that predominate in one subgroup over another.

As noted above, the model upon which screening recommendations rely conceives CRC as a disease that takes 10–20 years to develop. Evidence from the Minnesota Colon Cancer Control Study suggests that changes in screening rates can take over 10 years to have an impact on incidence rates [6]. If this lag time is correct, then changes in screening rates 1990–2004 should effect changes in incidence rates of cancer 2000–2014. The percentage of the general population undergoing colorectal screening in 1992 has been estimated at 25%[13], and it increased to approximately 50% by 2005 [14]. Recent studies have suggested that up to 60% of CRCs can be prevented by CRC screening, suggesting that at least some of the change in incidence rates 2000–2014 can be ascribed to increased colorectal screening [15,16]. Based on the predominant model of CRC progression (Fig 1), colorectal screening should prevent distant CRC more effectively than regional and localized disease. We conclude therefore that the relative stability of distant disease from 2000–2014 is not consistent with a screening effect. At the population level represented by SEER data, increases in CRC screening are associated with decreases in localized and regional disease, whereas increased screening has not been associated with the same amount of reduction in the incidence of distant disease. Given the success of CRC screening in reducing the incidence of CRC overall, we find this result both perplexing and disturbing. The analysis of SEER data presented here raises the question whether screening strategies can be modified in practical terms to reverse these trends [17,18].

### Inadequate screening does not explain the slow decrease in incidence of distant CRC

Why is the incidence rate of distant CRC not decreasing as quickly as expected? Could the slower decrease of distant CRC arise from inadequate screening? If the difference in rate decreases were due to inadequate screening, we would expect an association between differences in screening rates and differences in incidence rates. Because incidence and screening rates differ by race with whites having higher screening rates than non-whites [9,10], inadequate screening predicts that the decrease in incidence rate of distant disease in less frequently screening populations should be slower than in the higher frequency screening populations. Although the incidence of distant disease is higher in African Americans compared to whites, the rate of decrease in the incidence is similar between the two ethnic groups (Fig 3C), despite the fact that African Americans have until recently undergone CRC screening considerably less frequently than whites. The same trend is apparent in the Asian and Pacific Islander ethnic populations, which are populations that also have lower screening rates than whites. The finding that the slower decrease of distant CRCs is observed in a range of populations with different screening rates suggests that a defect in screening does not explain the incidence rate difference.

We considered whether tumor location might explain the slow decrease in distant CRCs because benign lesions are more likely to be missed in the proximal colon and adenocarcinoma is less likely to be detected by non-colonoscopic screening modalities. Going against this explanation, however, we found that the incidence rates of distant CRCs are decreasing at statistically slower paces compared to regional and localized disease among both proximal and distal CRCs. Thus, trends in distant CRC incidence rates are similar in both tumor locations (Fig 3B).

The lack of association between screening rates and incidence rates of distant CRC and between tumor location and incidence rates of distant CRC suggest that inadequate CRC screening is not sufficient to explain the slower decrease in the rate of distant disease, that is, it is not consistent with a screening effect.

### More rapidly advancing cancers of the serrated adenoma pathway can only explain part of the slow decrease in incidence of distant CRC

Most CRCs take a decade or more to develop to advanced stages, justifying the currently suggested screening intervals that are used to detect early lesions. CRCs that progress and metastasize quickly would therefore resist detection by screening because screening intervals are too long to prevent them.

There is a CRC subtype consisting of 10% to 15% of sporadic CRCs that is thought to develop more rapidly than the majority of CRCs, namely, CRCs that exhibit microsatellite instability (MSI) [19]. Sporadic CRCs that exhibit MSI develop predominantly due to methylation of the promoter region of mismatch repair gene *MLH1* [20–23]. Sporadic MSI CRCs are associated with a CpG island methylator phenotype, older age of diagnosis, and female sex [24]. They arise more frequently in the proximal colon, progress more rapidly [25], and are more likely to be diagnosed at a later stage [26]. There is evidence that tumors with MSI develop from flat lesions via mutation in *RNF43* (serrated adenoma pathway) rather than the adenoma to adenocarcinoma pathway, which is associated with gatekeeper mutations in the *APC* gene [27]. Moreover, CRC development in Lynch syndrome, which is associated with disease-causing mutations in mismatch repair genes, progresses more rapidly [28], leading to the recommendation of annual screening colonoscopy [29]. These data therefore suggest that screening is less effective for sporadic CRCs that develop via the serrated adenoma pathway. However, our analysis of SEER data showed that the decrease in incidence rate of distant CRCs was similar in the distal colorectum and in the proximal colon and there was no sex difference in the decrease in distant CRCs (Fig 3B & 3D), suggesting that CRCs of the MSI subtype are very likely not the sole explanation for the slower rate of decrease of distant cancers.

### Hypothesis of nonMSI CRC that advances rapidly

Our analysis has shown that the incidence of distant CRC is decreasing at a slower rate than localized and regional CRC, a trend which is not explained by a particular age group at diagnosis, tumor location, race, or sex. We propose that the difference in distant and non-distant CRC incidence reductions suggests the existence of rapidly advancing forms of CRC that develop and metastasize too quickly for screening to prevent them. Based on the considerations cited above, we hypothesize that there exists a microsatellite stable (i.e., nonMSI) CRC subtype that develops and progresses more rapidly than conventional CRC. Moreover, based on the more rapidly increasing incidence rate of distant CRC in the early-onset CRC age group, the unchanging incidence rate in the 50–64 age group, and the decreasing incidence rate in the 65+ age group, we suggest that this rapidly advancing cancer occurs more frequently in younger persons than in older persons.

Our hypothesis makes predictions that can be tested. First, the hypothesis predicts that a significant fraction of CRCs that present at a metastatic stage at initial diagnosis would be of the rapidly developing subtype. Consequently, we would expect cluster analysis of somatic mutations or expression profiles in these tumors to reveal either a distinct subtype or a significantly different distribution of known subtypes. Secondly, interval and early-onset cancers that present as metastatic CRCs should possess molecular signatures that are similar to those found in primary metastatic CRCs identified in non-screening patients. An important question is whether the molecular pathogenesis of the proposed aggressive nonMSI CRC subtype differs from the canonical adenoma-carcinoma sequence that is initiated by mutations of the *Adenomatous Polyposis Coli* (*APC*) gene, because this well-studied pathway is known to take 10 to 20 years to progress to CRC.

## Limitations and alternative theories

Limitations of the present study include the unavailability of information on whether CRC cases were screening, the frequency and modes of CRC screening, and clinical and molecular data such as mismatch repair gene immunohistochemistry. An important question raised by our analysis is whether the slower decrease in the incidence rate of distant CRCs could be explained by changes in exposures to the adverse lifestyle factors of diet and physical inactivity that are known to increase overall CRC risk, the times at which these exposures occur, or changes in usage of medications that reduce CRC risk, such as aspirin usage [30,31]. Unfortunately, it is not possible to assess the impact of exposures using SEER data [28,29].

As mentioned above, it is currently estimated that 60% of CRCs can be prevented by colonoscopy. Approximately three quarters of the CRCs that arise after a negative colonoscopy are due to missed lesions [32–34]. These considerations suggest that, even if all lesions were identified by colonoscopy, some fraction of interval cancers are new cancers. Importantly, it is difficult to distinguish missed lesions from newly developed cancers [35]. Although colonoscopy studies suggest that all stages of CRC are reduced by screening [7], the present study raises the question whether this conclusion is correct at the population level. Moreover, there is epidemiologic evidence that colorectal screening does not explain all of the reduction in CRC incidence since 1980 [36]. Consequently, the slower rate of reduction of distant cancers from 2000–2014 may be associated with a risk variable, such as low socioeconomic status, education, or medication history. In order to test this hypothesis, more comprehensive and individualized demographic and exposure data are needed.

Whatever the explanation for the differences in decrease of incidence rate by stage of presentation, a better understanding of the biology and epidemiology underlying this phenomenon is urgently needed. If CRC screening is truly ineffective in preventing metastatic disease at presentation, then biomarkers for possible early detection and targeted therapies for this type of metastatic disease are needed to improve survivorship of these patients. Further studies of CRCs that are metastatic at presentation would help us understand the biological and epidemiologic mechanisms underlying their occurrence and provide new approaches to impact the public health consequences of distant disease.

## Abbreviations

CRC: colorectal cancer
SEER: Surveillance, Epidemiology, and End Results
MSI: microsatellite instability
APC: *Adenomatous Polyposis Coli*
